# Bioactive Peptides from Animal By-Products: Production, Functional Evidence and Food Applications

**DOI:** 10.3390/foods15173143

**Published:** 2026-09-04

**Authors:** Ying-Yan Liang, Bo-Yu Cai, Li Chen, Gui-Can Bi, Jun Xie

**Affiliations:** Key Laboratory of Energy Plants Resource and Utilization, Guangdong Engineering Technology Research Center for Agricultural and Forestry Biomass, Ministry of Agriculture and Rural Affairs, College of Future Biomass, South China Agricultural University, Guangzhou 510642, China

**Keywords:** animal by-products, bioactive peptides, protein hydrolysates, structure-function relationships, functional food ingredients, food processing, upcycling

## Abstract

Animal-processing by-products contain collagen, myofibrillar proteins, blood proteins, whey proteins, and egg proteins that can be converted into peptide-rich food ingredients. Within a single application-oriented framework, this review integrates source heterogeneity, process control, peptide-profile characterization, tiered functional evidence, food-matrix performance, and regulatory substantiation. Evidence is evaluated for antioxidant, ACE-inhibitory, antimicrobial, DPP-IV-inhibitory, anti-inflammatory, mineral-binding, and taste-modulating functions, while distinguishing chemical assays, cell models, animal studies, human interventions, and tests in real-food matrices. Potential applications include functional foods, dietary supplements, natural preservation, flavor systems, texture modification, active packaging, and oral delivery. Translation remains limited by raw-material heterogeneity, batch variability, sensory defects, processing and gastrointestinal instability, uncertain bioavailability, incomplete safety assessment, and poorly defined regulatory claims. Future work should prioritize source traceability, peptide fingerprints, food-matrix validation, human exposure data, and scalable food-grade production. Compositionally defined peptide mixtures with reproducible functionality may be more practical than single highly purified sequences.

## 1. Introduction

### 1.1. Industrial Context and the Value of Animal By-Product Upcycling

Animal-based food processing generates substantial side streams from livestock and poultry slaughter, seafood processing, dairy manufacture, and egg processing [1,2,3,4,5,6]. Global meat production was estimated at about 365 Mt in 2024, while fisheries and aquaculture production reached 223.2 Mt in 2022 [1,2]. These figures demonstrate the scale of the processing systems, although they do not directly quantify the amount of by-products. Slaughter and meat-processing streams include bones, skin, tendons, blood, offal, fat, and trimmings, which contain collagen, myofibrillar proteins, plasma proteins, and other recoverable components [3,4]. Seafood streams include heads, bones, skin, scales, fins, viscera, and trimmings, many of which are rich in collagen, muscle proteins, minerals, or endogenous enzymes [2,5]. Whey, second cheese whey, residual egg white, and eggshell membrane add further protein-rich streams, but their use requires attention to composition, allergenicity, flavor, and regulatory status [6,7,8]. When these materials are relegated to low-value feed, fertilizer, or disposal, both protein value and potential functionality are lost, and the organic load of the processing chain remains high [3,5,6].

Converting suitable animal by-products into protein hydrolysates, bioactive peptides, collagen peptides, flavor bases, or functional ingredients can increase resource efficiency and product value, provided that safety and batch consistency are controlled [4,9]. The scientific rationale extends beyond replacing conventional protein sources. Controlled processing can release peptide populations with defined molecular features and measurable technological or biological functions. The central challenge is to connect raw-material quality and process conditions with reproducible peptide profiles and an application that is realistic for the food industry [9].

### 1.2. The Potential of Bioactive Peptides as Functional Food Ingredients

Bioactive peptides are short amino-acid sequences released from food proteins by enzymatic hydrolysis, microbial fermentation, gastrointestinal digestion, or related processing. Their behavior depends on sequence, molecular weight, hydrophobicity, charge distribution, conformation, and stability [10,11,12]. Animal proteins can yield peptide fractions with antioxidant, ACE-inhibitory, antimicrobial, immunomodulatory, metabolic, or mineral-binding activity [11,13]. Most reported effects originate from chemical assays, simulated digestion, cell models, or animal studies. These levels of evidence are not equivalent to efficacy in foods or humans.

### 1.3. Scope and Analytical Framework of This Review

**Figure 1:**
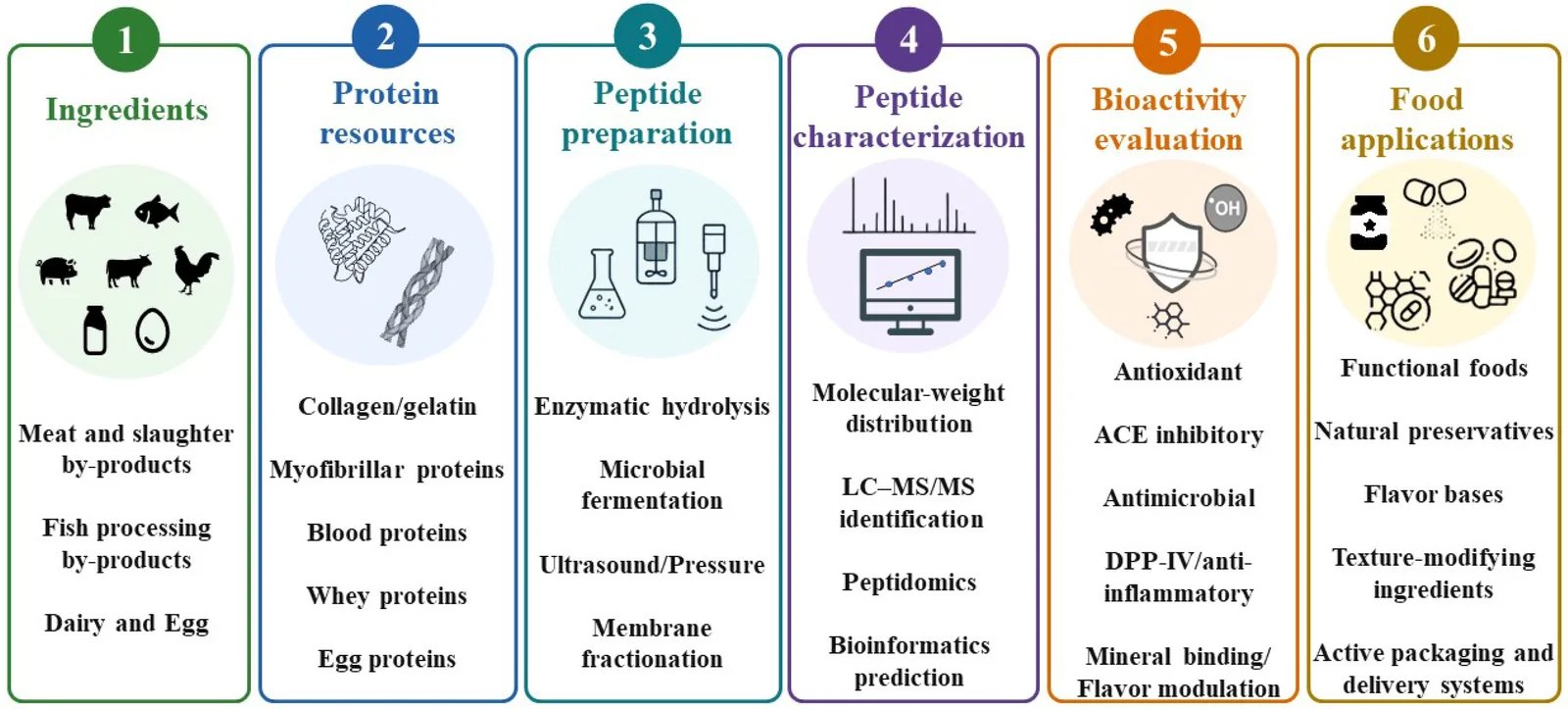
Integrated pathway from animal by-products to food-grade peptide ingredients.

This critical narrative review links animal by-product composition with peptide production, characterization, functional evidence, and food application (Figure 1). Recent reviews have provided valuable syntheses of meat-derived peptides; fish-processing by-products; broad food-derived peptide production and encapsulation; and regulatory, safety, and bioavailability issues [4,5,14,15,16,17]. These themes, however, are commonly addressed within separate source-specific, process-specific, or health-oriented frameworks (Table 1). The principal novelty of this review is its integration of source heterogeneity, process control, peptide-profile characterization, tiered functional evidence, food-matrix performance, and regulatory substantiation within a single application-oriented framework. The review compares protein resources from livestock and poultry, aquatic processing, dairy processing, and egg processing; evaluates hydrolysis, fermentation, process-intensification technologies, fractionation, mass spectrometry, peptidomics, and bioinformatic screening; and examines safety, sensory quality, scalability, quality control, and commercialization. The analytical pathway is therefore defined as raw-material suitability, controlled peptide release, structural characterization, tiered functional validation, food-matrix performance, and regulatory positioning.

### 1.4. Literature Search and Review Approach

The literature search was designed to support a critical narrative review rather than a formal systematic review or meta-analysis. Web of Science Core Collection, Scopus, PubMed, and Google Scholar were searched through July 2026. Search strings combined terms for the raw material (“animal by-product*”, “slaughter by-product*”, “meat by-product*”, “fish processing by-product*”, “seafood side stream*”, whey, “egg by-product*”, collagen, blood, bone, skin, and viscera) with terms for the product and process (“bioactive peptide*”, “protein hydrolysate*”, enzymatic hydrolysis, fermentation, ultrasound, high pressure, membrane filtration, chromatography, LC-MS/MS, peptidomics, bioinformatics, and molecular docking). Functional and application terms included antioxidant, ACE inhibitory, antihypertensive, antimicrobial, DPP-IV inhibitory, anti-inflammatory, mineral binding, umami, bitterness, functional food, preservation, edible coating, active packaging, and delivery.

Studies published mainly from 2020 to July 2026 were prioritized to reflect recent analytical, processing, and application advances. Earlier publications were retained when they provided foundational mechanisms, established methods, or evidence that remained directly relevant. Reference lists of recent reviews were also screened to identify primary studies. Priority was given to studies that clearly described the animal source and tissue, pretreatment or hydrolysis conditions, peptide fractionation or sequence-level characterization, and the experimental design used for functional validation. Studies reporting only a general activity value without adequate information on the material or process were used cautiously and were not treated as strong evidence for food application.

Evidence was interpreted by level rather than pooled quantitatively because materials, doses, assays, and endpoints were highly heterogeneous. Chemical radical-scavenging and enzyme-inhibition assays were considered screening evidence. Cell models were used to assess biological responses and mechanistic plausibility; animal studies were used to connect intake with physiological endpoints; and human interventions were considered the most relevant evidence for health-related claims. Tests performed in real-food matrices were evaluated separately because they establish processing, storage, sensory, oxidative, or microbial performance under application conditions. This hierarchy guided the discussion of claim strength, translational gaps, and research priorities.

Representative studies were interpreted against a tiered evidence framework rather than treated as equivalent forms of support (Table 2). Tier 1 covers chemical assays and enzyme-inhibition tests—useful for identifying activity, but insufficient to establish biological relevance. Cell models form Tier 2 and provide biological-response or mechanistic evidence, whereas animal studies (Tier 3) test whether an effect persists after administration. Tier 4 is reserved for human intervention studies, the most relevant evidence when a health-related claim is considered. Cell-based evidence is not scarce, but the models used vary considerably with the biological function under investigation. Common systems include intestinal epithelial-, vascular endothelial-, macrophage-, and metabolism-related cell models; in this review, these were discussed selectively when they provided a clear link between biochemical screening and physiological validation [13,15]. Real-food studies were kept as a separate application level because they address processing performance, stability, sensory properties, and technological behavior rather than biological efficacy.

## 2. Animal By-Products as Protein Sources for Bioactive Peptide Production

### 2.1. Livestock and Poultry Slaughter and Meat-Processing By-Products

Bones, skin, tendons, and cartilage are collagen-rich substrates for gelatin hydrolysates and collagen peptide ingredients. Blood provides hemoglobin, albumin, and plasma proteins that can yield antioxidant, antimicrobial, and metal-binding peptides. Meat trimmings and offal contain myofibrillar, sarcoplasmic, and connective-tissue proteins with potential for ACE-inhibitory, antioxidant, and flavor-active fractions [3,4,10]. These source-dependent opportunities also create distinct processing burdens. Blood requires decolorization and odor control; fatty tissues require efficient defatting and oxidation management; and offal and trimmings demand strict freshness and microbial control. A peptide process is only food-relevant when the starting material is traceable, stable, and compatible with the intended product.

### 2.2. Aquatic Processing By-Products

Aquatic processing produces skin, scales, bones, heads, fins, viscera, trimmings, and protein-containing process water. Solid by-products may account for roughly 55–65% of the catch weight, depending on species and processing route [14]. Their protein content and composition vary sharply by tissue: skin and scales favor collagen and gelatin production; bones combine collagen with calcium- and phosphate-rich mineral phases; and heads, viscera, and trimmings supply mixed muscle and connective-tissue proteins [16,22,23,24]. This diversity supports antioxidant, ACE-inhibitory, antimicrobial, mineral-binding, and flavor applications, but it prevents a single process from being applied indiscriminately. Lipid oxidation, fishy odor, bitterness, histamine and other biogenic amines, heavy metals, microorganisms, and allergens must be managed from raw-material collection onward [17,25]. Source-specific development is more defensible than treating all aquatic side streams as interchangeable peptide substrates [26].

### 2.3. Dairy and Egg-Processing By-Products

Whey and second cheese whey contain β-lactoglobulin, α-lactalbumin, lactoferrin, serum albumin, immunoglobulins, and glycoproteins. Their established food status, soluble protein fraction, and compatibility with beverages and dairy formulations provide a comparatively mature route to peptide ingredients [6,8,27]. Egg white, egg yolk residues, and eggshell membrane contain ovalbumin, ovotransferrin, ovomucoid, lysozyme, and structural proteins that can release antioxidant, ACE-inhibitory, anti-inflammatory, and metabolism-related peptides [7,28,29]. These streams are not risk-free. Whey hydrolysates often require bitterness control and stability testing, while egg-derived ingredients require allergen assessment and compliant labeling. Functional claims should progress from sequence identification and in vitro activity to digestion stability, cell or animal validation, and, where relevant, human evidence [27,29,30].

### 2.4. Blood-Derived Protein Ingredients and Heme Iron Valorization

Blood-derived proteins represent an important category of animal by-products with established nutritional and functional applications. Hemoglobin and globin fractions recovered from slaughterhouse blood can be processed into heme iron ingredients and bioactive peptide preparations. Heme iron exhibits high bioavailability compared with non-heme iron sources and has been widely investigated as a natural iron fortification ingredient [31]. In addition, enzymatic hydrolysis of hemoglobin and globin proteins can generate peptide fractions with antioxidant, antimicrobial, and other functional properties [32,33]. Therefore, blood-derived streams represent valuable resources for producing both nutrient-enhanced ingredients and bioactive peptide products, and should be considered alongside collagen-rich tissues, bone, and other animal by-products in value-added utilization strategies.

### 2.5. Source Protein Composition and Peptide Function

Source proteins define the sequence space available for proteolysis. Collagen is rich in Gly, Pro, and hydroxyproline and often produces small, imino-acid-rich peptides. Muscle proteins provide a broader distribution of the hydrophobic, aromatic, and charged residues associated with redox activity, ACE interaction, and taste. Blood proteins can yield potent peptide sequences but present additional color, flavor, and safety constraints. Milk and egg proteins have a substantial evidence base, yet the translation from enzyme or cell assays to human outcomes remains incomplete. A useful development model links source composition, cleavage specificity, peptide profile, mechanism and food-system performance rather than assigning functionality from source alone [13] (Table 3).

## 3. Production and Characterization Technologies

### 3.1. Enzymatic Hydrolysis and Microbial Fermentation

Enzymatic hydrolysis remains the most controllable route for producing food-grade peptide mixtures. Protease specificity, substrate concentration, enzyme-to-substrate ratio, pH, temperature, time, and degree of hydrolysis jointly determine molecular-weight distribution, sequence composition, yield, bitterness, and functionality [9]. Process optimization should not maximize hydrolysis indiscriminately. Excessive cleavage can destroy target sequences, increase free amino acids, weaken emulsifying or gelling behavior, and intensify bitterness. Fermentation releases peptides through microbial proteases and peptidases and may improve flavor or digestibility, but strain selection and matrix composition make the resulting peptide profile less predictable [36]. Enzymatic hydrolysis is generally better suited to standardized production; fermentation is particularly valuable when peptide release and flavor development are intended to occur together.

Acid-assisted hydrolysis is an established option for extensive protein breakdown, particularly in flavor-oriented hydrolysates. The process is comparatively simple and can achieve substantial conversion, but cleavage is less selective than in enzyme-controlled systems; peptide sequence and size distributions are consequently harder to tune. Acid treatment is better suited to broad hydrolysis than to the targeted release of defined bioactive sequences [37]. Combined acid-enzymatic hydrolysis has also been reported; in chicken-embryo proteins, conversion to hydrolysis products exceeded 60%, with a reported ≤3 kDa fraction yield of 93.7% [38].

Safety constraints become more important when acid treatment is combined with high temperature. Chloropropanols—including 3-monochloropropane-1,2-diol (3-MCPD) and 1,3-dichloropropanol (1,3-DCP)—are recognized process contaminants in acid-hydrolyzed protein products. Harsh conditions can also promote amino-acid degradation, racemization, and Maillard-type reactions, with consequences for both safety and sensory quality. Process design must balance hydrolysis efficiency against peptide quality and the formation of processing-derived products [39,40].

### 3.2. Emerging Processing Technologies for Assisted Hydrolysis

Ultrasound, high hydrostatic pressure, microwave treatment, pulsed electric fields, and subcritical water can alter protein structure, expose cleavage sites, and improve mass transfer before or during hydrolysis [9,41]. Their effects are matrix- and dose-dependent. Ultrasound can disrupt particles and unfold proteins through cavitation and shear [42]. High pressure modifies tertiary and quaternary structures but may also change aggregation or enzyme stability [43,44]. Pulsed electric fields can promote conformational changes and mass transfer, while subcritical water can hydrolyze proteins without conventional enzymes [42,45]. Higher peptide yield is not sufficient evidence of process improvement. Molecular-weight profiles, sequence retention, activity, bitterness, nutrient loss, energy demand, equipment cost, scale-up behavior, and batch reproducibility must be assessed together.

### 3.3. Isolation, Purification, and Structural Characterization

Animal-protein hydrolysates contain residual proteins, peptides of different sizes, free amino acids, salts, lipids, and other low-molecular-weight compounds. Ultrafiltration and nanofiltration are practical for high-throughput fractionation, but membrane fouling, peptide adsorption, selectivity, and recovery can limit performance [46]. Chromatography offers higher resolving power by size, charge, or hydrophobicity and is useful for activity-guided discovery, although solvent use, resin cost, and low throughput restrict routine industrial application. LC-MS/MS, MALDI-TOF-MS/MS, targeted quantification, and peptidomics can establish sequence-level evidence and peptide fingerprints [47]. Bioinformatic prediction and molecular docking narrow the candidate pool, but predicted activity, toxicity, allergenicity, bioavailability, and target binding require experimental confirmation [48,49]. For commercial ingredients, a reproducible peptide fingerprint may be more informative than isolating a single sequence that contributes little to the activity of the mixture (Table 4).

## 4. Major Bioactivities and Mechanisms of Action Related to Food Applications

Peptide ingredients are heterogeneous systems whose composition reflects the source protein, tissue structure, pretreatment, protease, degree of hydrolysis, fractionation, and food matrix. Collagen-rich tissues, mineralized tissues, blood, and muscle-derived streams do not generate equivalent peptide profiles. Mechanistic interpretation should begin with this compositional context rather than treating low molecular weight or high hydrolysis degree as universal indicators of activity [15,50].

Low molecular weight often coincides with stronger activity, but it is not an independent predictor of peptide function. Sequence, amino-acid composition, conformation, and bioavailability can outweigh size alone [51,52]. A fraction with a larger molecular-weight cut-off may retain—or even concentrate—more favorable sequences than the smallest fraction. Molecular-weight data should be interpreted together with peptide composition and activity profiles rather than used as a stand-alone proxy for potency. A direct counterexample was observed after sequential fractionation of chicken-embryo hydrolysates: the ≤3 kDa fraction accounted for 93.7% of the yield, yet the 10 kDa fraction showed the strongest ABTS-scavenging response [38].

Activity measured in a simplified assay may be lost during heating, storage, interaction with lipids or polysaccharides, gastrointestinal digestion, epithelial transport, or plasma metabolism [15,53,54]. Evidence should be interpreted in tiers: chemical and enzyme assays identify candidates; cell models test biological responses; animal studies connect exposure with physiological endpoints; and human interventions address relevance to intended use; real-food studies establish technological efficacy. Claims should not exceed the strongest level of evidence available.

**Figure 2:**
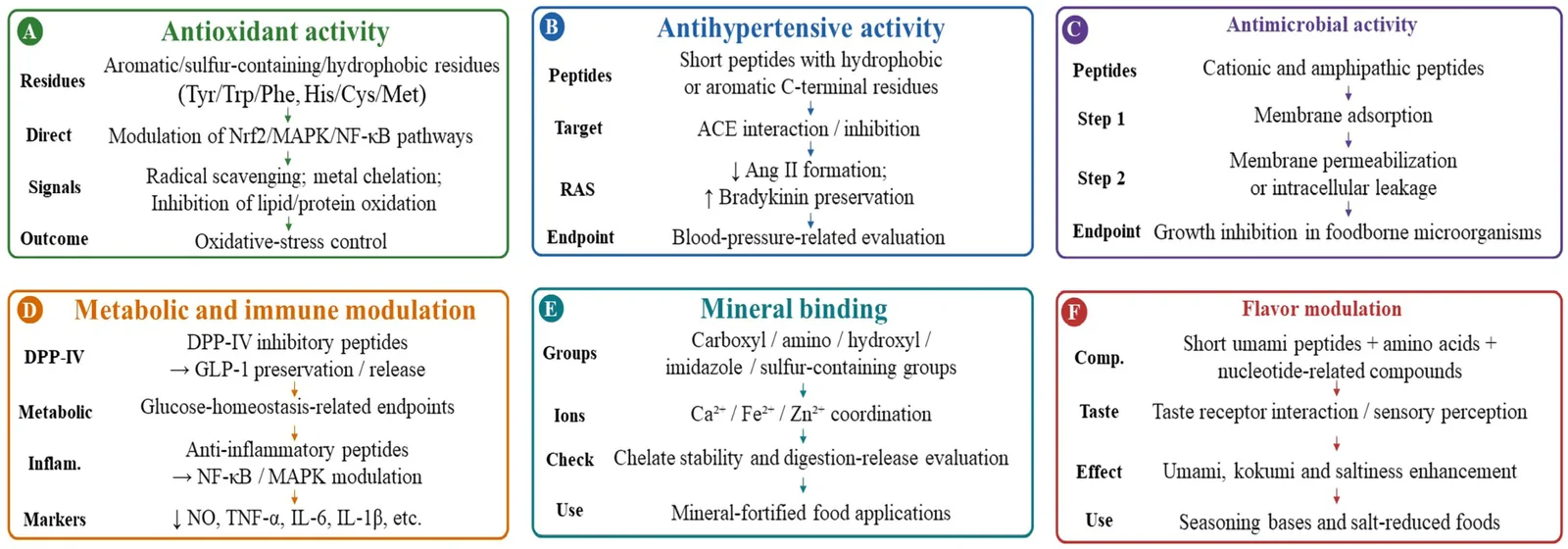
Major functions, mechanistic hypotheses, and validation endpoints for peptides derived from animal by-products.

The following sections examine antioxidant, ACE-inhibitory, antimicrobial, metabolic, immune-related, mineral-binding, and taste-modulating effects, together with safety considerations (Figure 2). Each function is discussed in relation to structural clues, appropriate endpoints, and the evidence still needed for food application.

### 4.1. Antioxidant Activity

Antioxidant peptides may scavenge radicals, chelate pro-oxidant metals, limit lipid or protein oxidation, or modulate cellular oxidative-stress pathways. Tyr, Trp, and Phe can stabilize radical species; His, Cys, and Met can participate in redox reactions or metal coordination; hydrophobic residues can promote interaction at lipid–water interfaces [55,56]. These relationships are probabilistic rather than deterministic because sequence order, terminal residues, conformation, concentration, and matrix partitioning also affect activity.

DPPH, ABTS, FRAP, and ORAC assays are useful for screening, but they do not establish oxidative protection in food or physiological antioxidant efficacy. Food-oriented validation should include lipid and protein oxidation markers, color and flavor stability, storage conditions, and dose–matrix interactions. Biological interpretation requires cellular or in vivo endpoints and evidence that active peptides or metabolites reach the relevant site.

Chemical antioxidant assays have assay-specific constraints that can distort comparisons between peptide preparations. DPPH is commonly run in alcoholic media, where some hydrophilic or higher-molecular-weight fractions disperse poorly and may yield a biased radical-scavenging response. Aromatic residues (Tyr, Trp, and Phe) can add absorbance or radical-quenching signals; Maillard products formed during processing can do the same, making it difficult to assign the response to peptides alone [57]. DPPH, ABTS, FRAP, and ORAC also probe different reaction chemistries—radical quenching, reducing power, or inhibition of peroxyl-radical oxidation—so their values are not interchangeable [58,59]. Results from any one of these assays are best treated as screening data and should be checked against food-matrix performance, cellular responses, or in vivo evidence before broader antioxidant claims are made.

Six antioxidant peptides were identified from tilapia-skin hydrolysate, and their radical interactions were explored by molecular docking [60]. Antioxidant activity was also demonstrated in chicken-feet protein hydrolysates [61], while five antioxidant peptides were identified from bovine-bone collagen hydrolyzed with recombinant collagenase [62]. These studies support sequence discovery but remain largely pre-application. Pork- and duck-skin gelatin hydrolysates were further evaluated in cooked sausage by monitoring lipid oxidation during refrigerated storage [63]. That matrix-based design provides stronger evidence for preservative use because it tests the ingredient under processing and storage conditions.

### 4.2. ACE-Inhibitory and Blood-Pressure-Related Effects

Most studies in this area begin with the inhibition of angiotensin-converting enzyme (ACE), which participates in angiotensin II formation and bradykinin degradation. Short chain length, C-terminal hydrophobic or aromatic residues, Pro position, charge, and conformation can influence interaction with the ACE active site [64,65]. ACE inhibition is a screening endpoint, not proof of a blood-pressure-lowering effect after ingestion.

Peptide release from tilapia-skin type I collagen during Alcalase hydrolysis showed that cleavage kinetics and Pro position influence ACE-inhibitory fractions [18]. A combination of ultrafiltration, peptidomics, molecular docking, and endothelial-cell testing was used to identify ACE-inhibitory candidates from eel-bone collagen [19]. Evidence from bovine-bone gelatin was further extended from in vitro ACE inhibition to blood-pressure endpoints in animals [20]. Together, these studies illustrate a progression from process kinetics and sequence discovery to biological validation.

Orally consumed peptides may be cleaved during digestion, transported intact or as metabolites, degraded in plasma, or fail to reach systemic targets. A credible evidence chain should therefore include simulated digestion, intestinal transport or permeability, exposure measurements, animal blood-pressure endpoints, and controlled human intervention where a health claim is intended [56,66].

### 4.3. Antimicrobial Activity

Food-derived antimicrobial peptides are often cationic, amphipathic, and moderately hydrophobic, properties that favor adsorption to microbial membranes and may lead to permeabilization, leakage, or intracellular disruption [67,68]. Matrix composition can markedly reduce activity through ionic strength, fat binding, proteolysis, pH, or adsorption to other food components. Inhibition zones and minimum inhibitory concentrations are preliminary measures. Minimum bactericidal concentration, time-kill kinetics, biofilm assays, food challenge tests, storage studies, and sensory evaluation are needed before preservative use can be supported.

The bovine-hemoglobin-derived antimicrobial peptide neokyotorphin (NKT; α137–141) was enriched by ultrafiltration [69]. Its recovery during electrodialysis–ultrafiltration was influenced by the degree of hydrolysis [70]. Carp-skin gelatin hydrolysates were incorporated into a chitosan-red-algal-polysaccharide coating [71], and a fish-skin gelatin hydrolysate coating was evaluated on refrigerated shrimp [72]. The latter studies are more directly relevant to food preservation because they test microbial and quality outcomes in applied systems.

These peptides are more plausible as components of hurdle preservation, edible coatings, or active packaging than as stand-alone replacements for refrigeration, sanitation, or conventional preservation. Application studies should also monitor microbial ecology, resistance selection, food-contact safety, flavor, and the stability of activity during storage.

### 4.4. Metabolic Regulation and Immune-Related Activities

Metabolic studies frequently target dipeptidyl peptidase IV (DPP-IV), whose inhibition can prolong incretin activity, including glucagon-like peptide-1 (GLP-1). Whether an inhibitory peptide remains active depends on digestion, intestinal residence, absorption, and the concentration reached at the target [73]. Anti-inflammatory studies commonly measure NO, PGE_2_, TNF-α, IL-1β, IL-6, NF-κB, or MAPK responses [74]. Changes in these markers support a mechanistic hypothesis but do not by themselves demonstrate a clinically relevant effect.

DPP-IV-inhibitory peptides were prepared from proteinase K hydrolysates of donkey-blood hemoglobin, and the <3 kDa fraction reduced fasting blood glucose, improved glucose tolerance, partially restored liver and pancreatic morphology, and modulated gut microbial composition in type 2 diabetic mice [75]. The DPP-IV-inhibitory peptides GPF, IGL, and GGGW were identified from chicken-blood hydrolysates [76]. Yak-bone collagen peptides were associated with reduced NO production and NF-κB-related responses [77], while a sturgeon-cartilage hydrolysate was evaluated in LPS-stimulated RAW264.7 macrophages [78]. The evidence is promising but heterogeneous in material, dose, model, and endpoint, which limits direct comparison.

A defensible interpretation separates enzyme inhibition, cell signaling, animal physiology, and human outcomes. Without human data, these ingredients should be described as candidates for metabolic-health or inflammatory-homeostasis applications rather than as agents that prevent or treat disease.

### 4.5. Mineral-Binding and Flavor-Modulation Functions

Peptide ligands containing carboxyl, amino, hydroxyl, imidazole, or sulfur-containing groups can coordinate Ca^2+^, Fe^2+^, Zn^2+^, and other minerals [34,79]. Binding capacity alone does not establish nutritional benefit; chelate stability, release during digestion, competition with other food components, and mineral absorption must also be measured. Taste-active peptides can contribute umami [61,80], kokumi-like richness [18,62,63], or saltiness enhancement [18,19,20]. Their value depends on sensory threshold, receptor interaction, mixture effects, and behavior in the finished formulation [80,81].

Phosphate-bearing residues provide an additional class of mineral-binding sites that is not captured by carboxyl, amino, or hydroxyl groups alone [35]. Casein phosphopeptides (CPPs) contain clusters of phosphoserine residues with high affinity for calcium and other minerals. By maintaining soluble peptide-mineral complexes, these motifs can influence mineral solubility and intestinal availability—a key reason CPPs remain among the best-studied food-derived mineral-binding peptides [34,35]. Phosphorylation status should be considered when the mineral-binding potential of a peptide ingredient is characterized.

A one-pot process was developed to release fish-scale peptides, calcium, and phosphate during pepsin hydrolysis [22]. Bovine-bone collagen peptide–calcium chelates were characterized, and low-molecular-weight tuna-bone collagen peptide–calcium complexes were further examined [82,83]. These studies establish binding and stability, but bioavailability remains the decisive nutritional endpoint. In flavor research, umami peptides were identified in beef-bone soup, while peptides from fermented goose bones were characterized using sensory and receptor-based approaches [84,85]. Mineral-binding peptides should focus on verifying chelation stability, digestive release, and mineral utilization effects; flavor peptides should focus on verifying sensory thresholds, receptor action, bitterness control, and flavor contributions within the formulation system (Table 5).

### 4.6. Safety and Sensitization Assessment

Edible origin does not guarantee the safety of a peptide ingredient. Assessment should cover the source material, collection and storage, processing aids, ingredient composition, exposure, and the final food. Relevant hazards include pathogens, spoilage, biogenic amines, veterinary-drug residues, heavy metals, environmental contaminants, animal-health risks, residual enzymes, lipid-oxidation products, process-induced reaction products, and source-specific allergens [21,86,87]. Molecular-weight distribution, total peptide content, free amino acids, salt, ash, and characteristic peptide markers are also needed to define the ingredient and demonstrate batch consistency. For compositional specification, total peptide content can be quantified using an OPA-based spectrophotometric assay, whereas free amino acids can be determined chromatographically after appropriate derivatization [88,89,90].

A randomized crossover study comparing plasma amino acids, dipeptides, and tripeptides after intake of fish-, porcine-, and bovine-derived collagen hydrolysates showed that source and peptide profile can alter postprandial exposure [85]. Extraction, pretreatment, and hydrolysis may also generate or retain allergenic, toxic, or otherwise undesirable components [86]. Processing can modify fish-protein antigenicity—reducing, preserving, or exposing allergenic determinants depending on the treatment [87]. Safety conclusions must therefore be ingredient-specific rather than inferred from the parent protein.

Risk characterization should define the animal source, tissue, manufacturing process, composition, intended dose, and target population. Aquatic materials, blood, offal, and lipid-rich streams need particularly strict control of freshness, oxidation, environmental contaminants, pathogens, and cross-contamination. Human tolerability and allergen assessment become more important as intake increases or products target vulnerable populations (Table 6).

Repeated dietary exposure raises safety questions that are not captured by microbiological, contaminant, allergen, or compositional testing alone. Normal-cell cytotoxicity assays can assess cellular compatibility, while transformed-cell models can reveal whether a preparation alters proliferative behavior. Mutagenicity or antimutagenic screening adds another layer of evidence when peptides are positioned for tissue-repair or regeneration-related functions [38,91]. These assays do not replace conventional safety tests—they complement them by probing biological responses that composition alone cannot predict. A recent study of chicken-embryo hydrolysate fractions illustrates the point [38]. Alongside antioxidant, anti-inflammatory, and wound-healing outcomes, the authors tested cytotoxicity in normal fibroblasts, proliferation in HeLa and Vero cultures, and antimutagenic activity. The fractions were non-cytotoxic and did not stimulate cell growth in those cultures, showing why multifunctional peptide ingredients may require a broader safety panel than routine compositional specifications.

## 5. Food-Industry Applications

**Figure 3:**
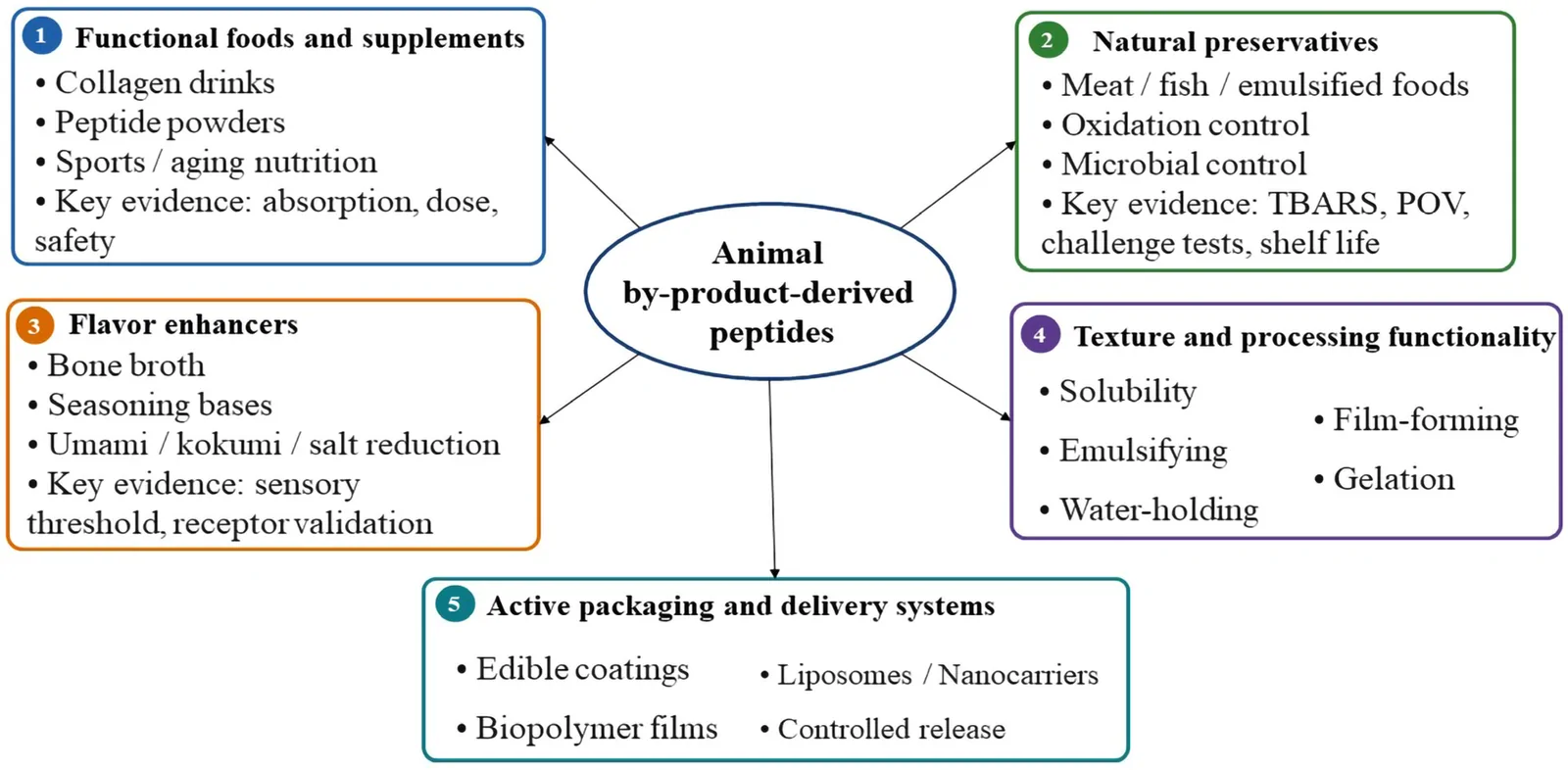
Principal food applications and application-specific evidence requirements.

Application determines the evidence required (Figure 3). Functional foods and supplements depend on ingredient identity, safety, dose, bioavailability, and human relevance. Preservation and active-packaging applications require oxidation, microbial, and shelf-life outcomes in real foods. Flavor and texture applications are judged primarily by sensory contribution, formulation compatibility, processing stability, and cost. A single activity result cannot substantiate all of these applications.

### 5.1. Functional Foods and Dietary Supplements

Hydrolyzed collagen is among the most established animal-derived peptide ingredients because it is soluble, processable, and readily incorporated into beverages, powders, dairy products, nutrition bars, sports-nutrition products, and foods for older adults [50]. Other animal by-product hydrolysates may enter similar formats, but their commercial readiness depends on composition, sensory quality, safety, and regulatory status rather than molecular weight alone.

The evidence chain should be matched to the proposed function. Collagen products require characterization of absorbed peptides or metabolites, a justified dose, and relevant human endpoints. DPP-IV-inhibitory products require evidence beyond enzyme inhibition, including glycemic outcomes, tissue responses, and, where relevant, gut-microbiota changes in appropriate animal models [75]. Ingredients positioned around inflammatory homeostasis need exposure data and appropriately controlled biological models [77].

The principal gap is the limited number of well-controlled human studies with defined peptide profiles, dose–response analysis, and long-term safety. In practice, raw-material continuity, specifications, palatability, compatibility with the food format, and a viable claim pathway can be as decisive as biological activity [92].

Hydrolyzed collagen is commercially mature, but market presence should not be conflated with the strength of evidence behind a physiological claim. Reported effects vary with peptide composition, molecular-weight distribution, dose, study design, and the endpoint being measured. Even when cell, animal, and human data point in the same direction, differences in formulation and exposure can limit direct comparison. Functional positioning is stronger when the product is chemically defined and supported by well-designed human intervention studies at a justified dose. In the EU, an Article 13(5) joint-health claim for collagen hydrolysate was not substantiated; the EFSA concluded that a cause-and-effect relationship had not been established [93,94].

### 5.2. Natural Antioxidants and Natural Preservatives

Animal by-product hydrolysates can be evaluated as antioxidant or antimicrobial components in meat, seafood, emulsions, and lipid-containing foods. The realistic aim is to slow lipid and protein oxidation, color loss, flavor deterioration, or microbial growth as part of a hurdle system. These ingredients should not be presented as substitutes for sanitation, thermal processing, cold-chain control, or appropriate packaging.

The use of bioactive peptides and protein hydrolysates in meat preservation has been reviewed [95]. Pork- and duck-skin gelatin hydrolysates were evaluated in cooked sausage [63]. A peptide-containing edible coating was tested [71], and food-grade enrichment of a hemoglobin-derived antimicrobial peptide was investigated [69]. These studies illustrate complementary requirements: ingredient preparation must be reproducible, and preservative performance must be demonstrated in the target food during storage.

Complex composition can produce batch variation, bitterness, color changes, off-odors, and matrix-dependent effects. Applied studies should report dose, matrix composition, processing, storage temperature, oxidation indices, microbial counts, color, texture, sensory quality, and interaction with other preservation hurdles.

### 5.3. Flavor Enhancers and Seasoning Bases

Protein hydrolysates from bone, meat trimmings, and connective tissue can provide meaty, umami, rich, or salt-enhancing notes in soup bases, seasoning powders, flavor concentrates, and reduced-sodium foods [80,89,96]. Unlike health-positioned products, these ingredients are judged mainly by sensory performance, formulation synergy, process tolerance, and price.

Umami peptides were characterized in beef-bone soup [84]. Peptides from fermented goose bones were isolated and their interactions with the T1R1/T1R3 receptor were examined [85]. Virtual screening, sensory testing, and molecular docking were combined to identify umami peptides from porcine type I collagen [97]. These approaches improve candidate selection, but receptor binding and model-solution thresholds must still be confirmed in complex foods.

Development should include sensory thresholds, descriptive analysis, bitterness and off-odor control, thermal stability, salt-reduction performance, and consumer testing. The contribution of an individual sequence should also be distinguished from the combined effect of peptides, free amino acids, nucleotides, salts, and aroma compounds in the hydrolysate [97].

### 5.4. Food Texture Modification and Functional Applications in Processing

Hydrolysis changes solubility, emulsification, foaming, water-holding capacity, interfacial behavior, film formation, and gelation. These properties depend on molecular-weight distribution, hydrophobicity, charge, conformational flexibility, and aggregation [98,99]. The desired profile is application-specific: beverages require solubility and clarity; meat products require water retention and thermal stability; films and gels require cohesive networks and acceptable mechanical properties.

Gelatin and gelatin hydrolysates obtained from extrusion-pretreated tilapia scales were compared, showing that processing altered molecular-weight distribution, physicochemical properties, and antioxidant performance [100]. A fish-gelatin-fucoidan edible film was characterized [101], while synergistic water retention among peptide fractions was demonstrated in a thermally processed meat model [102]. These results show that peptide fractions can be technological ingredients, not only carriers of biological activity.

The degree of hydrolysis must be optimized for the target food. Extensive cleavage often improves solubility but can weaken gel formation, interfacial films, and mechanical strength while increasing bitterness. Functional testing should therefore be performed at the intended pH, ionic strength, temperature, concentration, and processing history.

### 5.5. Active Packaging and Delivery Systems

Antioxidant or antimicrobial hydrolysates can be incorporated into edible films, coatings, protein films, polysaccharide matrices, or composite packaging. Surface localization may reduce the amount of active ingredient required and limit effects on bulk flavor and texture. Performance depends on compatibility with the polymer network, release kinetics, barrier properties, mechanical integrity, and activity throughout storage [103,104].

Peptide-containing biopolymer films have been systematically reviewed [103]. A chitosan-polysaccharide coating containing carp-skin gelatin hydrolysate was tested [71], while a fish-skin gelatin hydrolysate coating was evaluated on whole shrimp [72]. These studies connect material properties with food quality and microbial outcomes, providing a stronger basis for packaging claims than activity assays alone.

For orally consumed products, liposomes, emulsions, nanoparticles, hydrogels, and protein–polysaccharide systems may protect peptides or modify their release [105,106,107] (Table 7). Encapsulation efficiency is only an initial metric. Digestion, release, bioaccessibility, processing tolerance, storage stability, sensory effects, safety, and regulatory acceptability must also be evaluated. Salmon by-product peptides were encapsulated in marine liposomes, while nanoliposomes were used to protect cod-derived osteogenic peptides [106,107].

## 6. Commercialization Challenges and Research Priorities

### 6.1. Raw Material Safety and Quality Control

Species, tissue, age, rearing conditions, slaughter, storage, and pretreatment alter protein composition, lipid content, mineral content, contamination risk, and the peptide profile obtained after hydrolysis. Variability is particularly high among aquatic skin, scales, heads, viscera, bones, and trimmings [108]. Raw-material classification and acceptance specifications should precede enzyme optimization; otherwise, process consistency cannot compensate for uncontrolled input variability.

Quality control should extend from source traceability and cold-chain records to microbial and contaminant limits, protein composition, hydrolysis parameters, molecular-weight distribution, peptide fingerprints, and application-relevant activity. A characteristic peptide or multipeptide fingerprint can link raw-material batches with process performance and functional consistency, provided that the marker is stable, analytically measurable, and related to the ingredient rather than an incidental trace component [86].

Process integration was demonstrated in one-pot fish-scale peptide–calcium production [22]. Hydrolysis degree and membrane or electroseparation conditions were shown to determine the recovery of hemoglobin-derived antimicrobial peptides [69,70]. Release kinetics, rather than a single end-point ACE assay, were used to characterize tilapia-collagen hydrolysis [18]. These examples support process analytical control instead of reliance on final-product testing alone.

**Figure 4:**
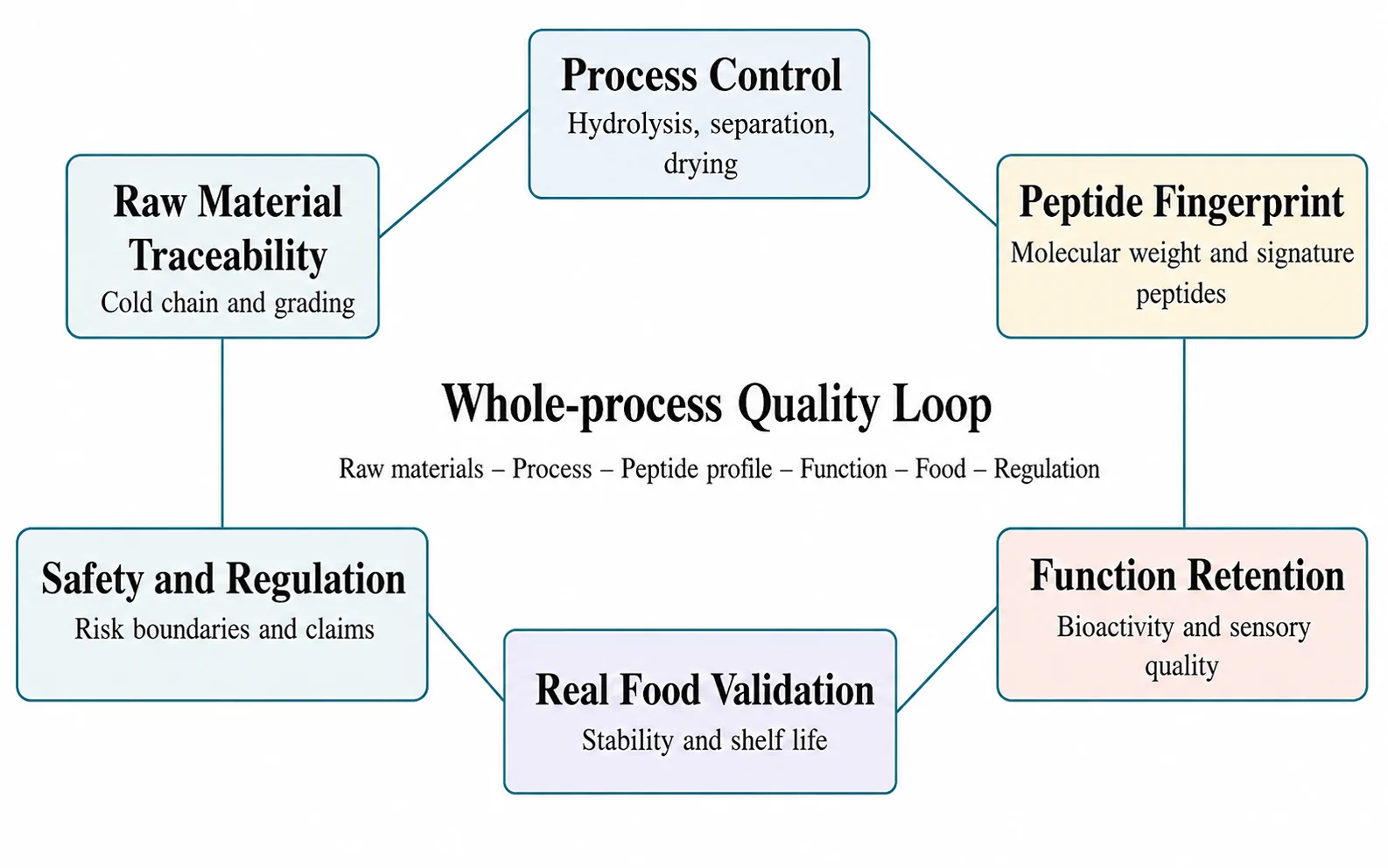
Closed-loop quality control from raw-material traceability to claim substantiation.

A closed-loop system should connect raw-material records, hydrolysis and separation data, drying and storage conditions, peptide-profile specifications, and functional retention (Figure 4). Shared analytical standards and databases would also improve comparability between studies, which is currently weakened by differences in substrates, enzymes, units, and activity assays.

### 6.2. Sensory Quality and Consumer Acceptance

Bitterness, fishy or gamey odor, metallic notes, dark color, and an overly strong broth profile can restrict use in beverages, dairy products, ready-to-eat foods, and premium functional products [81,96]. Sensory limitations are not secondary formulation issues; they determine the maximum usable dose and may prevent an ingredient from reaching the level required for a claimed function.

Hydrophobic short peptides are often associated with bitterness, and more extensive hydrolysis can increase their release even when solubility improves [96]. Fishy and gamey odors may arise from oxidized lipids, volatile amines, sulfur compounds, and source-specific aroma precursors. Control should begin with fresh raw materials and defatting, then combine enzyme selection, fermentation, fractionation, debittering, deodorization, encapsulation, and formulation masking as needed.

Thermal processing was shown to generate desirable taste-active peptides in beef-bone soup [84]. Fermentation was used to develop flavor-active fractions from goose bones [85], while sensory and receptor-based evidence was combined for porcine-collagen umami peptides [97]. These studies suggest that flavor development and off-flavor control should be designed together rather than treated as separate downstream problems.

Acceptance also depends on terminology, animal origin, price, perceived naturalness, sustainability claims, cultural and religious considerations, and trust in the stated benefit. Studies of upcycled foods show that product quality, health and environmental information, technology aversion, and labeling influence willingness to try or purchase [109,110,111]. Product communication must be accurate enough to avoid overstating both sustainability and health value.

### 6.3. Processing Stability, Bioavailability and Efficacy

Peptides may be hydrolyzed, oxidized, aggregated, bound by the food matrix, or sterically masked during manufacture, storage, and digestion. Low molecular weight does not guarantee absorption or efficacy. Orally consumed peptides encounter gastric acid, digestive enzymes, mucus, the intestinal epithelium, plasma peptidases, and first-pass metabolism [66,90]. Some functions may be exerted locally in the gastrointestinal tract, whereas systemic claims require evidence of exposure at a relevant concentration.

Source- and molecular-weight-dependent differences in postprandial peptide exposure were demonstrated after collagen hydrolysate intake [21]. A broader evidence chain for donkey-blood hemoglobin peptides linked DPP-IV inhibition with reduced fasting blood glucose, improved glucose tolerance, partial restoration of liver and pancreatic morphology, and modulation of gut microbial composition in type 2 diabetic mice [75]. Such studies are more informative than activity screening alone, yet they still require replication with defined peptide profiles, justified doses, and relevant human endpoints.

A staged strategy is practical: screen stability under standardized digestion, examine transport or local intestinal activity, quantify circulating peptides or metabolites where appropriate, and then test functional endpoints in animals and humans. Linking measured exposure with an observed effect would substantially strengthen causal interpretation and dose selection.

Translation from an in vitro signal to a physiological effect is constrained by exposure. Enzyme-inhibition and antioxidant assays often use concentrations chosen to produce a measurable response under simplified conditions, whereas oral intake subjects peptides to digestion, absorption barriers, enzymatic degradation, and systemic metabolism. The circulating concentration of an intact or still-active fragment can be much lower than the concentration used to derive an in vitro IC_50_. This exposure gap—not the IC_50_ alone—should guide interpretation of physiological relevance. Claims based on in vitro potency need support from bioavailability or pharmacokinetic measurements and, where appropriate, in vivo validation [30,111]. A useful scale comparison is provided by two studies discussed here: after 10 g porcine collagen hydrolysate, Pro-Hyp reached a ΔCmax of 3.8 μg/mL (approximately 17 μM) in plasma [21], whereas the donkey-blood DPP-IV-inhibitory fraction had an in vitro IC_50_ of 1.95 mM [75]—about two orders of magnitude higher. The peptide systems are not directly matched, so this comparison is illustrative rather than pharmacokinetic proof.

### 6.4. Regulatory Status and Claim Substantiation

Food-chain eligibility comes before questions of claim wording or novel-food status. Slaughter and processing streams are heterogeneous: some tissues may be suitable for food use, whereas others require exclusion or specific controls. A food-grade peptide process begins with the legal category of the raw material, traceable origin, compliant processing establishments, and confirmation that the selected tissue is permitted for the intended food use [15,91,112]. An edible source animal does not automatically confer the same regulatory status on every by-product derived from it.

Recent reviews of food-derived bioactive peptides and functional foods show that regulatory classification varies markedly among jurisdictions and governs source approval, safety substantiation, manufacturing controls, labeling, and the types of claims that may be used [15,113]. Regulatory assessment must be market-specific; a history of consumption for the parent animal or protein does not automatically establish the status of a concentrated hydrolysate.

Claim strength should follow evidence strength. In vitro findings support statements about measured activity; cell and animal studies can support mechanistic or physiological plausibility; well-designed human studies are generally required for claims about benefits in people. Without human evidence, wording should remain at the level of potential application or candidate functionality. Disease-prevention or treatment language is inappropriate for conventional food ingredients.

The specification should identify species, tissue, processing method, composition, molecular-weight profile, relevant peptide markers, contaminants, allergens, microbiological limits, oxidation indicators, and intended intake. These data are needed even when the source animal is edible because concentration and processing can change exposure and risk.

Ruminant-derived materials carry an additional regulatory burden because TSE controls—most notably those developed for BSE—affect the use of bovine, ovine, and caprine tissues in collagen and gelatine production. Risk assessments consistently treat tissue origin, exclusion of specified risk materials (SRMs), and compliance with validated processing requirements as central safety determinants. For bovine- and ovine-derived peptide ingredients, this means documented tissue selection, controlled sourcing, and validated processing before food use is considered [93,94].

### 6.5. Future Research and Industry Development Directions

Research priorities should be organized around a connected evidence pathway rather than further expansion of peptide inventories. Candidate sequences identified by peptidomics, bioinformatics, docking, or machine learning should be verified through quantitative analysis, standardized digestion, transport or local intestinal models, target-relevant assays, and application-specific testing. Integrated studies such as kinetic peptide-release analysis [18], fractionation coupled with peptidomics and biological validation [19], and sensory screening linked to receptor analysis [97] illustrate this direction.

For product development, priority should be given to reproducible raw-material specifications, peptide fingerprints, processing and storage stability, sensory acceptability, effective intake, safety, cost, and regulatory feasibility. Shared reference materials, harmonized analytical units, and real-food validation would improve cross-study comparability. These priorities provide the basis for the industrialization challenges summarized in Table 8 and for the broader future directions discussed below.

## 7. Conclusions and Future Directions

Animal by-products should not be treated as interchangeable protein reservoirs. Species, tissue, physiological state, handling, and pretreatment determine the starting proteome, while protease specificity and downstream fractionation determine which sequences are released and retained. The resulting ingredient is therefore the outcome of a source-process-composition continuum rather than a generic “peptide powder.” This perspective helps explain why hydrolysates with similar average molecular weights can differ substantially in biological activity, sensory quality, processing stability, and safety. Future studies should report raw-material provenance, process history, degree of hydrolysis, quantitative molecular-weight distribution, and a reproducible peptide fingerprint as a minimum characterization package.

The main scientific gap is no longer a shortage of candidate activities but a weak causal chain connecting an identified peptide or peptide mixture to a meaningful effect in food or in vivo. Chemical assays and molecular docking can prioritize candidates, but neither establishes efficacy. Stronger studies should integrate quantitative peptidomics with standardized digestion, intestinal transport or local gastrointestinal action, target engagement, dose–response behavior, and application-specific endpoints. When systemic action is proposed, circulating peptides or metabolites should be measured wherever technically feasible. Human trials should use chemically defined batches, justified doses, appropriate comparators, and outcomes that match the intended claim. Negative and null findings are also valuable because they define the limits of activity and reduce repeated screening of candidates that are unlikely to translate.

Translation will require an ingredient-development strategy built around predefined specifications rather than retrospective description. Raw-material traceability, contaminant control, oxidation management, allergen assessment, batch-to-batch peptide markers, sensory thresholds, processing stability, and shelf-life performance should be incorporated from the beginning. Validation in real foods is essential because pH, salts, lipids, polysaccharides, heat, and storage can alter peptide accessibility and function. A purified peptide is not automatically the most practical product. When activity depends on complementary sequences, a compositionally controlled multipeptide fraction may offer greater robustness and lower manufacturing cost, provided that marker peptides and functional acceptance criteria are defined. Claims of by-product valorization should also be supported by mass balance and life-cycle evidence, since water-intensive processing or extensive purification can erode the expected sustainability benefit.

Several priorities could accelerate progress. Shared reference materials and interlaboratory protocols are needed for molecular-weight analysis, peptide quantification, bioactivity assays, digestion models, and reporting units. Peptide databases should incorporate source tissue, processing conditions, abundance, stability, matrix performance, and validated biological endpoints rather than sequence-activity annotations alone. Artificial intelligence and multi-omics can help integrate these data and identify response patterns, but their outputs should remain testable hypotheses. Regulatory planning should proceed in parallel with product development so that safety studies, human evidence, and claim wording are matched to the intended market. The field will mature when success is measured not by the number of newly reported sequences, but by the delivery of reproducible, safe, sensorially acceptable, and economically viable peptide ingredients whose benefits remain demonstrable in foods and, where claimed, in humans.

## Figures and Tables

**Table 1:** Comparison of recent reviews and the positioning of the present review.

Scope	Main Emphasis	Positioning Relative to the Present Review
Meat and meat by-products [4]	Extraction, activities, applications, and limitations of meat-derived peptides.	Source-specific synthesis; limited integration of other animal streams, real-food performance, and regulation.
Fish-processing by-products [5]	Extraction, non-thermal processing, bioavailability, safety, and food applications.	Aquatic focus; cross-source process control, peptide fingerprints, and claim substantiation are less central.
Food-derived bioactive peptides [13]	Extraction, purification, biological functions, and encapsulation across food proteins.	Broad peptide-technology coverage; not centered on animal by-products or end-to-end industrial validation.
Food-derived bioactive peptides [15]	Regulation, safety, digestion, absorption, and bioavailability.	Detailed on translation; less emphasis on source classification, process reproducibility, sensory quality, and real-food testing.
Fishery by-product hydrolysates [16]	Production, composition, technological properties, food applications, and industrial challenges.	Strong fish-hydrolysate framework; limited cross-source comparison and tiered functional evidence.
Fish protein hydrolysates [17]	Nutritional, bioactive, technological, safety, and food-application aspects.	Links fish hydrolysates with foods; broader animal sources, peptide-profile quality control, and claim substantiation remain outside its main scope.
Present review	Animal sources, controlled production, peptide profiles, evidence levels, food applications, safety, sensory quality, scale-up, and regulation.	Cross-source framework linking source-process-composition relationships with evidence tiers, real-food performance, quality control, and commercialization.

**Table 2:** Evidence hierarchy applied for evaluating bioactive peptide functionality.

Evidence Level	Experimental Approach	Interpretation in This Review
Tier 1	Chemical assays, antioxidant assays, enzyme inhibition assays	Preliminary screening of potential activity [18]
Tier 2	Cell-based models	Biological response and mechanistic plausibility [19]
Tier 3	Animal studies	Physiological relevance after intake [20]
Tier 4	Human intervention studies	Evidence supporting health-related claims [21]
Application level	Real-food matrix validation	Processing performance, stability, sensory and technological applicability

**Table 3:** Animal by-products, major protein resources, and development priorities.

Source	Typical By-Products	Major Proteins	Principal Development Routes	Key Food-Development Considerations
Livestock and poultry slaughter/meat processing	Bones, skin, tendons, cartilage, blood, offal, fat, and trimmings	Collagen, myofibrillar proteins, blood proteins (hemoglobin and globin proteins)	Collagen peptides; antioxidant, ACE-inhibitory, antimicrobial, metabolism-related fractions; heme iron ingredients and globin-derived bioactive peptide fractions	Defatting, decolorization, odor control, freshness, contaminants, allergenicity, heme stability, iron oxidation, and batch consistency [3,4,9,15,31,32,33]
Aquatic processing	Skin, scales, bones, heads, fins, viscera, and trimmings	Collagen, muscle proteins, minerals	Antioxidant, ACE-inhibitory, antimicrobial, mineral-binding, and flavor-active fractions	Lipid oxidation, biogenic amines, environmental contaminants, fishy odor, allergens, and cold-chain control [2,5,14,16,22]
Dairy processing	Whey, second cheese whey, and milk-protein side streams	Whey proteins, casein and casein-derived phosphopeptides	Antioxidant and ACE-inhibitory fractions; metabolic applications; nutritional fortification; mineral-binding casein phosphopeptides (CPPs)	Solubility, bitterness, digestion stability, allergens, regulatory status, phosphorylation pattern and mineral-binding performance [6,8,13,34,35]
Egg processing	Egg-white and egg-yolk residues, eggshell membrane, and related streams	Ovalbumin, ovotransferrin, ovomucoid, lysozyme, membrane proteins	Antioxidant, ACE-inhibitory, anti-inflammatory, and metabolism-related fractions	Allergenicity, egg odor, ingredient specifications, and formulation compatibility [7,28,29]

**Table 4:** Production and characterization technologies for food-grade peptide ingredients.

Processing Stage	Typical Methods	Primary Functions and Outputs	Industrial Limitations
Raw-material pretreatment	Cleaning, defatting, decolorization, and desalting	Reduces interference from lipids, pigments, salts, microorganisms, and off-odors; improves substrate processability	Waste-stream treatment, protein loss, contaminant control, and preservation of freshness [3,4,5,6,7,8,9]
Hydrolysis/fermentation	Food-grade proteases, enzyme combinations, lactic acid bacteria, and other fermentation systems	Releases peptide populations and controls molecular weight, sequence composition, degree of hydrolysis, and flavor	Protease specificity, bitterness, batch reproducibility, enzyme cost, and scale-up or continuous processing [43,44]
Process intensification	Ultrasound, high hydrostatic pressure, microwave treatment, pulsed electric fields, and subcritical water	Promotes protein unfolding, cleavage-site exposure, and mass transfer	Equipment investment, energy use, localized overprocessing, nutrient loss, and scale-up [41,42,43,44,45]
Separation and purification	Ultrafiltration, nanofiltration, size-exclusion chromatography, and ion-exchange chromatography	Enriches fractions according to molecular weight, charge, or hydrophobicity	Membrane fouling, recovery, solvent and resin cost, selectivity, and throughput [46,47,48]
Structural characterization	LC-MS/MS, peptidomics, targeted quantification, bioinformatics, and molecular docking	Identifies sequences, supports peptide fingerprints, and generates structure-function hypotheses	Predictions require experimental validation; databases, reference standards, and quantitative methods remain incomplete [49]
Acid-assisted/combined acid-enzymatic hydrolysis	Protein substrates are hydrolyzed under acidic conditions, either alone or followed by enzymatic hydrolysis, to generate peptide-rich hydrolysates	Simple operation, high hydrolysis efficiency, and established industrial applicability for flavor-oriented hydrolysates	Limited control of peptide sequence distribution; potential amino acid degradation, racemization, and formation of process-related contaminants [37,39,40]

**Table 5:** Major functions, structural clues, evidence boundaries, and food applications.

Functional Focus	Structural or Mechanistic Clues	Common Evaluations and Evidence Boundaries	Food Applications
Antioxidant	Aromatic and sulfur-containing residues; moderate hydrophobicity; radical scavenging, metal chelation, and oxidative-stress modulation	DPPH, ABTS, FRAP, and ORAC support screening; lipid/protein oxidation, cell responses, and real-food performance require separate validation	Lipid-containing foods, meat products, and active packaging [52,53,60,61,62,63]
ACE inhibition/blood-pressure-related effects	Short sequences; C-terminal hydrophobic or aromatic residues; Pro position; charge and conformation	ACE inhibition is not equivalent to reduced blood pressure; digestion, transport, exposure, animal endpoints, and human evidence are needed	Candidate functional-food ingredients [18,19,20,64,65,66]
Antimicrobial	Cationic, amphipathic, and hydrophobic sequences; membrane interaction and intracellular disruption	MIC/MBC and time-kill assays are initial tests; food challenge studies, biofilm assays, shelf life, and sensory effects are needed	Hurdle preservation, edible coatings, and active packaging [67,68,69,70,71,72]
Metabolic and immune related	DPP-IV inhibition and GLP-1 response; NO, TNF-α, IL-1β, IL-6, NF-κB, and MAPK	Enzyme inhibition, cell responses, animal physiology, and human outcomes must be reported as distinct evidence levels	Candidate ingredients for metabolic-health and inflammatory-homeostasis applications [73,74,75,76,77,78]
Mineral binding and flavor	Metal-coordinating groups; taste-active sequences; receptor interactions and mixture effects	Mineral applications require stability, release, and absorption; flavor applications require sensory thresholds, bitterness control, and formulation testing	Mineral-fortified foods, soup bases, seasonings, and reduced-sodium foods [34,79,80,82,83,84,85]

**Table 6:** Safety and quality-control matrix for peptide ingredients derived from animal by-products.

Control Stage	Key Risks	Recommended Control Targets
Raw materials and cold chain	Spoilage, pathogens, biogenic amines, animal disease, and uncertain origin	Species and tissue traceability, temperature records, microbial limits, pathogen testing, and biogenic amines
Contaminants and residues	Heavy metals, environmental contaminants, veterinary-drug residues, and lipid-oxidation products	Risk-based contaminant and residue panels; peroxide value or other oxidation markers
Manufacturing process	Acid/base damage, excessive heat or hydrolysis, enzyme residues, and Maillard-reaction products	pH, temperature, time, enzyme specifications, molecular-weight distribution, and process-induced products
Process-induced contaminants	3-MCPD, 1,3-DCP, amino-acid racemization, and Maillard-reaction products generated during harsh acid/thermal processing	Targeted monitoring of chloropropanols and other process-derived products; control of acid concentration, temperature, treatment time, and subsequent purification
Composition and consistency	Batch variation, undefined peptide populations, and changes in salt or ash	Total peptides (OPA spectrophotometric assay), free amino acids (RP-HPLC/DAD after pre-column derivatization), characteristic peptide fingerprint, salt, ash, and molecular-weight distribution
Digestion, exposure, and allergenicity	Changes in antigenicity, sensitizing peptides, uncertain systemic exposure, and excessive intake	Standardized digestion, allergen assessment, exposure or plasma-peptide analysis where relevant, tolerability, and justified dose limits
Biological safety	Potential cytotoxicity, abnormal cell proliferation, and mutagenic effects during long-term exposure	Cytotoxicity assessment using normal cells; evaluation of proliferation responses in transformed cell models; mutagenicity/antimutagenic screening when appropriate

**Table 7:** Food-application pathways and application-specific evaluation criteria.

Application Pathway	Typical Products or Systems	Key Performance Indicators	Principal Challenges
Functional foods and dietary supplements	Collagen-peptide beverages, dairy products, nutrition bars, and peptide powders	Defined composition, effective dose, digestion stability, exposure, safety, and human endpoints	Evidence level, claim boundaries, long-term safety, palatability, and regulatory status [21,50,75,77]
Flavor enhancers and seasoning bases	Soup bases, seasoning powders, meat flavors, and reduced-sodium foods	Umami or richness thresholds, descriptive sensory analysis, off-flavors, and formulation synergy	Complex mixture effects, debittering, deodorization, heat processing, and consumer acceptance [80,81,84,85,96,97]
Texture and processing functions	Beverages, dairy products, meat products, baked foods, gels, and edible films	Solubility, emulsification, foaming, water retention, film formation, gelation, and interfacial stability	Excessive hydrolysis may impair gelation or film formation and increase bitterness [98,99,100,101,102]
Active packaging and delivery	Films, coatings, liposomes, emulsions, nanoparticles, and hydrogels	Release, barrier/mechanical properties, digestion stability, processing tolerance, food-contact safety, and bioavailability	Encapsulation efficiency is not efficacy; sensory, stability, safety, and regulatory criteria remain essential [71,72,103,104,106,107]

**Table 8:** Challenges to industrialization and priority development strategies.

Challenge	Key Reasons	Priority Strategy
Raw-material and batch variation	Species, tissue, age, rearing, slaughter, storage, and pretreatment differences	Traceability, grading, compositional specifications, molecular-weight profiles, and peptide fingerprints [86,108]
Sensory defects and acceptance	Bitterness, fishy/gamey odor, metallic notes, color, animal origin, and upcycled-food perceptions	Defatting, decolorization, enzyme selection, fermentation, fractionation, encapsulation, formulation, and consumer testing [81,96,109,110]
Stability and bioavailability	Processing, storage, digestion, aggregation, oxidation, and matrix binding	Staged digestion–transport–animal–human validation and correlation of exposure with functional endpoints [21,53,66]
Evidence and claim boundaries	In vitro effects cannot directly substantiate human benefit or disease-related claims	Match wording to evidence level and establish dose–response, human relevance, and safety thresholds [15,113]
Scale-up and cost	Yield, capital expenditure, membrane fouling, energy demand, and incomplete standards	Food-grade continuous processing, quality markers, mass balance, and functional-stability control [16,17,22,46,47,48,49]

## Data Availability

No new data were created or analyzed in this study. Data sharing is not applicable to this article.
